# *Pandemica Panoptica*: Biopolitical Management of Viral Spread in the Age of Covid-19

**DOI:** 10.1007/s11196-021-09821-1

**Published:** 2021-02-04

**Authors:** Anne Wagner, Aleksandra Matulewska, Sarah Marusek

**Affiliations:** 1 ULR 4487 - CRDP - Centre de recherche Droits et Perspectives du droit, University of Lille, 59000 Lille, France; 2 Adam Mickiewicz University, Poznań, Poland; 3 University of Hawai‘I Hilo, Hilo, Hawai‘i USA

**Keywords:** SARS-CoV-2 pandemic, Covid-19, Discipline, Punish, Bodies, Mobility, Immobility, Modern panopticon

## Abstract

The current pandemic period has triggered a series of changes in society, at both individual and collective behavioral levels. These changes were perceived as either positive or negative by the impacted bodies, leading to both social change and positive interactions in a tense context. In this paper, the authors will deal with *Pandemica Panotpica*, subjugation infiltrating all levels of society, and the approach adopted by several countries in trying to find countermeasures to combat the virus' proliferation. Our research scope began at the onset of the pandemic and ended on early January 2021.

## Power Over Life: Political Discourse of Discipline

On December 2019, a newly identified severe acute respiratory syndrome coronavirus 2 (SARS-CoV-2) emerged in Mainland China [9], in Wuhan province, with people having fever, cough, and often pneumonia. It resulted in a fast outbreak throughout the province, and the authorities decided to lock down the city in order to stop its propagation. Unfortunately, this virus, which is now known to be extremely pathogenic, has had enough time to travel beyond the borders imposed by the Chinese government, and has thus spread invisibly throughout the world, creating major movements of concern among the people, despite attempts to put the situation into perspective. The predicament very quickly got out of hand with overwhelmed health authorities looking for ways to thwart the virus’ advance while waiting for a possible, very distance availability of a vaccine. As a result, some countries took radical decisions to contain SARS-CoV-2 by closing their borders and quarantining entire peoples with very significant restrictions and constraints on their mobility and/or immobility. Some countries, such as France through its President, have spoken of a war against the virus, a “health war”. This war economy was therefore set up to protect the people and fight against this invisible scourge, which is still little known and whose short-, medium- and long-term consequences are still being investigated.

As scientific knowledge about the virus evolved, this war economy consisted in giving high priority to the *first front line* by trying to provide them with all the necessary means to combat but also to protect them. Since China was closed, it was necessary to take over, and so ephemeral production lines appeared to build artificial respirators, to produce hydroalcoholic solutions, facemasks and any other kind of protection against SARS-CoV-2. Famous perfume, alcohol, petroleum and motor factories temporarily transformed themselves and took over to create the products needed to combat this virus. Even some concert, exhibition or sport halls were transformed into temporary units with volunteers to produce protective masks, first for the population at risk and the health community. Similarly, field hospitals were organized in tents surrounding hospitals, in exhibition and sports halls in Poland, Spain and Italy for instance. In Italy and Spain, there was also a need to convert ice-rinks to temporary morgues, since there was not enough space with controlled temperature, to store corpses until burial at the peak of death toll. Even in France, the Rungis market near Paris was partly converted into a mortuary due to lack of space. The *second front line* comprised liberal practitioners. They had to implement new protocols to protect not only themselves but also their patients. Remote consultations were given high priority to avoid the displacement of potentially contaminated patients. The *third front line* was the population on which the government imposed severe restrictions on movement, except for priority reasons, in case teleworking could not be implemented.

These different front lines in combating SARS-CoV-2 thus emphasize body politicization, its controls and impositions. It is the most modern version of Foucault’s panopticon, where discipline is the dominant principle and where body is under coercion means, “making those on whom they are applied clearly visible” [5, p. 171]. Somehow, the main impact of the panopticon consists in inducing in citizens’ minds “a state of conscious and permanent visibility that assures the automatic functioning of power” [5, p. 201]. So, power can only be exercised when bodies are alive, meaning that death is its limit [3, p. 138]. That is the reason why countries set a map of body’s mobility/immobility where innumerable points of confrontation were highly considered, like instability, risk of conflict and struggle. This common matrix implemented a temporary though strong inversion of power relations, as the State had an invisible control over citizens’ bodies, within either the public sphere or the private domain. The State therefore intruded into the domestic environment in an elusive way, resulting in greater control of bodies, similar to the prison system dealt by Foucault [5]. Indeed,

[…] power is not exercised simply as an obligation or a prohibition on those who “do not have it”; it invests them, is transmitted by them and through them; it exerts pressure upon them, just as they themselves, in their struggle against it, resist the grip it has on them [5, p. 27]

Thus, through the media, the State reinforced subjugation on citizens to exert an “influence on life, that endeavors to administer, optimize, and multiply it, subjecting it to precise controls and comprehensive regulations” [3, p. 137], esp. in information and communication technologies [7]. These supervisions were implemented in myriads of ways, (1) with advertising messages over a short time period aimed at monitoring our cleaning and disinfection habits and compliance with travel restrictions and social distancing, (2) with weekly health assessment to show the situation worldwide and in the country, sometimes with the intervention of medical experts, Health Ministers among others. The chief purpose of deploying such communication means was to instill in inhabitants’ minds notions of common sense, but also to regulate and appease residents in order to avoid any friction, and somehow with an undisguised desire to make citizens aware of the risks involved if they do not comply with the government regulations, not only for themselves but also for the vulnerable people around them.

In our paper, the modern panopticon in SARS-CoV-2 pandemic relates to subjugation, “[infiltrating] every level of society as all manners of individuals to question how to best govern themselves, their families and even their souls” [5, pp. 229–230]. The notion of “deprivation” viewed by Foucault [4], has undergone here a profound transformation to administer, secure and develop a politics of protection against SARS-CoV-2 taking into account mobility, immobility as well as the psychological impacts on bodies over this particular time period, and so:

deduction has tended to be no longer the major form of power but merely one element among others, working to incite, reinforce, control, monitor, optimize, and organize the forces under it: a power bent on generating forces, making them grow, and ordering them, rather than one dedicated to impeding them, making them submit, or destroying them. [3, p. 136]

## Disciplining, Partitioning, and Punishing Individual Bodies

Lockdowns were enforced at some point when the pandemic was said to be uncontrollable. So, bodies were manipulated to activate precise controls and regulations over them, in a power relation producing and sustaining a “system of constraints and privations, obligations and prohibitions” [3, p. 11]. From there on, governments no longer needed to use the concept of the panopticon, as initially thought by Bentham [2] with a large watchtower in the center of a circular prison [112], but they imposed an “economy of suspended rights [3, p. 11], where “the separations should be clear and the openings well arranged” [3, p. 202], being “a figure of political technology” [3, p. 205]

This economy consisted in having compulsory visibility on individual bodies within specific spaces, for determined periods of time and precise mobilities. It is still effective nowadays, with fewer limitations that may evolve if SARS-CoV-2 proves to be increasingly active in spaces. Therefore, the manipulation over individual bodies is highly important, not only outside but also inside of public and/or private spaces, generating a new mode of surveillance over inhabitants. This new power distribution results in (1) *the scale of control*, (2) *the object of control*, and (3) *the technique of control*, a “codification that partitions as closely as possible time, space, movement” [5, p. 137], enabling individual body control, even at distance. This panoply of controls subjugates individual bodies, and enforces proper ways of body movement or retreat, as dictated by the appropriate regulating authorities within determined spatial and temporal framework(s):

[It includes] all devices that [are] used to ensure the spatial distribution of individual bodies (their separation, their alignment, their serialization, and their surveillance) and the organization around those individuals, of a whole field of visibility [6, p. 242]

In France, severe restrictions on individual body movement were imposed on 23 March 2020 [14], which prescribed the general measures to deal with SARS-CoV-2 pandemic in a state of public health emergency, article 8 [14], namely.

1. Displacement to make purchases of supplies necessary for the professional activity and purchases of basic necessities,
2. Displacement between home and the place of professional activity, when the exercise of activities cannot be organized in the form of telework or professional travel cannot be postponed,
3. Consultations and care that cannot be provided at a distance and cannot be postponed,
4. Consultations and care for patients with long-term illnesses,
5. Travel for urgent family reasons, for assistance to vulnerable persons or for childcare,
6. Short displacements, within the limit of one hour per day and within a maximum distance of one kilometer from home, and
7. Judicial or administrative summons.

Each time a French citizen travelled, during the lockdown period, he/she had to fill in and carry at any time a duly completed and signed certificate of derogatory travel.^1^ If he/she breached these travel restrictions, the citizen could be subject to a lump-sum fine, and if he/she repeated the same offence the citizen could even be placed in prison.^2^ As data on SARS-CoV-2 pandemic evolved, France implemented other decrees [11–13] to enable better control of the situation while allowing the country's economic activity to resume and a greater mobility of individual bodies, thus increasing and restraining individual freedom at the same time.

In England, lockdown laws were enforced with significant restriction on people’s free movement by prohibiting leaving home without a “reasonable excuse” under the Health Protection (Coronavirus, Restrictions) (No. 2) (England) Regulations 2020, section 8 [26]:

- **8.**—(1) A person who without reasonable excuse contravenes a requirement in regulation 4, 5, 6(10), (11) or 7 commits an offence.
- (2) A person who obstructs, without reasonable excuse, any person carrying out a function under these Regulations, including any person who is a relevant person for the purposes of regulation 7, commits an offence.
- (3) A person who, without reasonable excuse, contravenes a direction given under regulation 7, or fails to comply with a reasonable instruction or a prohibition notice given by a relevant person under regulation 7, commits an offence.
- (4) An offence under this regulation is punishable on summary conviction by a fine.
- (5) If an offence under this regulation committed by a body corporate is proved—
- (a)to have been committed with the consent or connivance of an officer of the body, or.
- (b)to be attributable to any neglect on the part of such an officer,
- the officer (as well as the body corporate) is guilty of the offence and liable to be prosecuted and proceeded against and punished accordingly.

English citizens were restricted on gatherings either within the public and private spheres under the Health Protection (Coronavirus, Restrictions) (N°2) (England) Regulations 2020, section 5 [27]:

- **5.**—(1) During the emergency period, unless paragraph (3) applies, no person may participate in a gathering which—
- (a)consists of more than thirty persons, and
- (b)takes place—
- (i)in a private dwelling, including a houseboat,
- (ii)on a vessel, other than a houseboat or a vessel used for public transport, or
- (iii)on land which satisfies the condition in paragraph (2).
- (2) Land satisfies this condition if it is a public outdoor place, which is not—
- (a)operated by a business, a charitable, benevolent or philanthropic institution or a public body as a visitor attraction, or
- (b)part of premises used for the operation of a business, charitable, benevolent or philanthropic institution or a public body. [27]

As the situation evolves, so do the restrictions within the four nations (England [28], Scotland [23], Wales [22], and Northern Ireland [29]) with various ways of gathering, of using face coverings in public. These restrictions were also implemented with local lockdown laws, in Leicester [25] and in North of England [24]. The government also created the “Eat Out to Help Out” scheme to sustain the economic resumption following SARS-CoV-2 lockdown [86]. As from September 14, 2020, new rules apply with the concept of “support bubble” [58],^3^ meaning it will be against the law to meet people who do not live with in a group larger than 6. Police will then have the power to enforce these legal limits, with fines of £100, doubling for further breaches up to a maximum of £3200.

In the Republic of Poland, school classes were suspended on 12th March till summer holidays. The period from March till the end of pandemic is marked by the wave of regulations enacted by various ministers limiting the liberties of citizen in the effort to curb the pandemic spread. The Regulation of the Minister of Health of 24 March 2020 [18] amending the regulation on declaring an epidemic in the territory of the Republic of Poland turned out to be especially controversial. It stated that:

§ 3a. 1. In the period from 25 March 2020 till 11 April 2020, the movement of persons staying in the territory of the Republic of Poland is prohibited, except for the movement of a given person for the purpose of:

1) performing professional activities or official tasks, or non-agricultural economic activity, or conducting agricultural activity or work on an agricultural holding, and the purchase of related goods and services;

2) satisfying the necessary needs related to the current affairs of everyday life, including obtaining health or psychological care, of the person, of the person closest to him or her within the meaning of art. 115 § 11 of the Act of 6 June 1997 - Penal Code (Journal of Laws of 2019, items 1950 and 2128), and if the moving person stays in cohabitation with another person - also the person closest to the person remaining in the joint living, and the purchase of related goods and services;

3) voluntarily and without remuneration to provide benefits to counteract the effects of COVID-19, including as part of volunteering services;

4) exercising or participating in religious worship, including religious activities or rites.

2. Where the movement takes place:

1) on foot - two people may move at the same time at a distance of not less than 1.5 m from each other;

2) by means of collective public transport within the meaning of art. 1a paragraph 4 point 3a of the Act of 20 June 1992 on the entitlement to concessionary journeys by means of collective public transport (Journal of Laws of 2018, item 295) - this means transporting, at the same time, no more people than half the seats.

At the same time during the performance of religious worship, including religious activities or rites, in a given area or in a given facility, there could be a total of, both inside and outside the premises, no more than the total of 50 people and 5 participants, except for religious persons or persons employed by a funeral firm in the event of a funeral.

From 25 March 2020 until 11 April 2020, it was forbidden to move around Poland due to the COVID-19 coronavirus epidemic. The restrictions did not include a few exceptions, such as commuting to work, purchasing goods and services related to professional activities, or going out to satisfy the essential needs of everyday life such as buying alimentary, medical or hygiene products. It also did not apply to people who volunteered to fight COVID-19 (this applies to helping people in quarantine or people who should not leave home). One could also leave home to visit a doctor or take care of relatives. People living in cities suffered most as they were not allowed to go to parks, forests and other green areas. That placed a heavy strain on them. The online battle of hate speech ensued as the doubts concerned the exemptions, that is to say people exempt from the restrictions who were considered unlawfully privileged. For instance, the ban on visiting forests was criticized as not applicable to hunters combating the African Swine Fever epidemic. As a result of the heavy criticism, the hunters were also forbidden to enter forests. The question soon followed whether they were allowed to go out to guard the crop fields or whether the immobility order encompassed not only forests, parks and beaches but also agricultural areas.

In the Republic of Poland, the Regulation of the Council of Ministers of 31 March 2020 [15] on the establishment of certain restrictions, orders and bans in connection with an epidemic was enacted [20]. In the Regulation we read:

§ 2. 1. In the period from 31 March 2020, until further notice, the movement of passengers in rail transport that cross the border of the Republic of Poland shall be suspended. [20]

The people exempt from the ban included: crews of aircraft, ships, trains, fishermen, professional drivers, soldiers, diplomats and workers of diplomatic missions. At the same time the Regulation introduced ban on exporting respirators and other medical equipment. It also forbade larger meetings of various types and introduced limits for religious ceremonies, weddings and wedding receptions, concerts, sports events, etc. The next enacted regulations included (1) the Regulation of the Council of Ministers of April 1, 2020 [16, 19] amending the regulation on the establishment of certain restrictions, orders and bans in connection with the occurrence of an epidemic [21] and (2) the Regulation of the Council of Ministers of April 7, 2020 [17] amending the regulation on the establishment of certain restrictions, orders and bans in connection with the occurrence of an epidemic [21]. The Polish government announced the limits of 5 persons not living under the roof of the host to participate in Xmas and travelling restrictions are to be implemented to keep the citizens in their hometowns. The national quarantine was prolonged till the end of January 2021 and the police hours were introduced for the night of New Year’s Eve.

In the U.S., domestic restrictions on travel are determined by the individual states. In the contiguous states, travel between states that border each other is more difficult and determined by license plate. Crossing state lines in states that have emergency proclamation-based quarantines in effect can result in fines [143]. Even in the non-contiguous states of Hawaii and Alaska, quarantines are in effect to curb the travel of potentially infected bodies. However, no one state within the United States can ban travelers from other states, as federal mandates of travel take precedence over individual state restrictions. Nonetheless, each state’s quarantine is intended to limit travel, even among its own residents who venture beyond their state’s borders.

Consequently, regulating authorities have tried and are still trying to discipline and punish individual bodies, especially if they do not comply with the regulations that have been/are imposed to prevent further SARS-CoV-2 spread. These partitioned individual bodies tend to maximize their utility, docility or even opposition against regulating authorities. Individual bodies are vehicles of power, but power is inextricably intertwined with knowledge:

Knowledge linked to power, not only assumes the authority of 'the truth' but has the power to make itself true. All knowledge, once applied in the real world, has effects, and in that sense at least, 'becomes true.' Knowledge, once used to regulate the conduct of others, entails constraint, regulation and the disciplining of practice. Thus, 'there is no power relation without the correlative constitution of a field of knowledge, nor any knowledge that does not presuppose and constitute at the same time, power relations. [5, p. 27]

From this policy of imposed discipline(s), within society different population reactions have emerged, which highlight three types of individual bodies: useful, docile, and conflicting individual bodies.

### Useful Individual Body

When SARS-CoV-2 pandemic broke out around the world and de facto paralyzed the national economies, the different regulatory authorities decided to give high priority to the bodies that were useful to the nation and formed the front line against the pandemic. These useful individual bodies were, at first, essentially composed of health care professionals who were the ones to handle the situation to their best knowledge at that time, to provide treatments to sick bodies and to try and find medical or even technical solutions that could benefit their patients.

After a few weeks of hard struggle, the medical staff was joined by others to find technical solutions and provide extra hands to treat more and more sick bodies in increasingly overcrowded hospitals. Liberal nurses, doctors, vets, retired medical staff, medical students joined the ranks. Companies came together, providing equipment and facilities to help find appropriate solutions. Engineers and students helped them by developing and transforming everyday objects into useful devices that could replace the increasingly visible shortages in the fight against the virus. For this group of useful individual bodies, regulations were more flexible and allowed them to intervene more quickly without having to permanently provide a temporary *laissez-passer* (France) [90]. These useful individual bodies came together to intensify the fight and find short-term solutions in record time, while waiting for medical experts to establish comprehensive strategies for treating patients even better.

In France, one category of useful individual bodies was called “la reserve sanitaire” [52], which was quickly mobilized for short missions with a view to reinforcing the supply of health care or medico-social services. Even the armed forces healthcare was engaged to provide assistance with the setting up of military field hospitals to alleviate the congestion in public hospitals. The French Air Force and the Armed Forces Health Service implemented an A330 MRTT Phoenix [83], equipped with the “Morphée” module,^4^ to transfer patients suffering from SARS-CoV-2 from one country to another. In addition, a specially set-up TGV high speed train was used to evacuate patients to other parts of France. Consequently, the country put itself in motion to fight against SARS-CoV-2 with all the means at its disposal.

The obsession of useful individual bodies resulted in the creation of a specific discourse oriented towards them so as to pay a tribute to them in public [127, 128]. People, from celebrities to ordinary citizens, ended up forming a chain of hope around them, encouraging them with songs [80, 87, 88], financial aids [109, 36], preparing and delivering meals to them by chefs [34, 56], and/or simply by honking horns or applause at certain times of the day. These crowd movements had the effect of boosting their morals, showing them gratitude, and encouraging them at all costs [129].

**Fig. 1:**
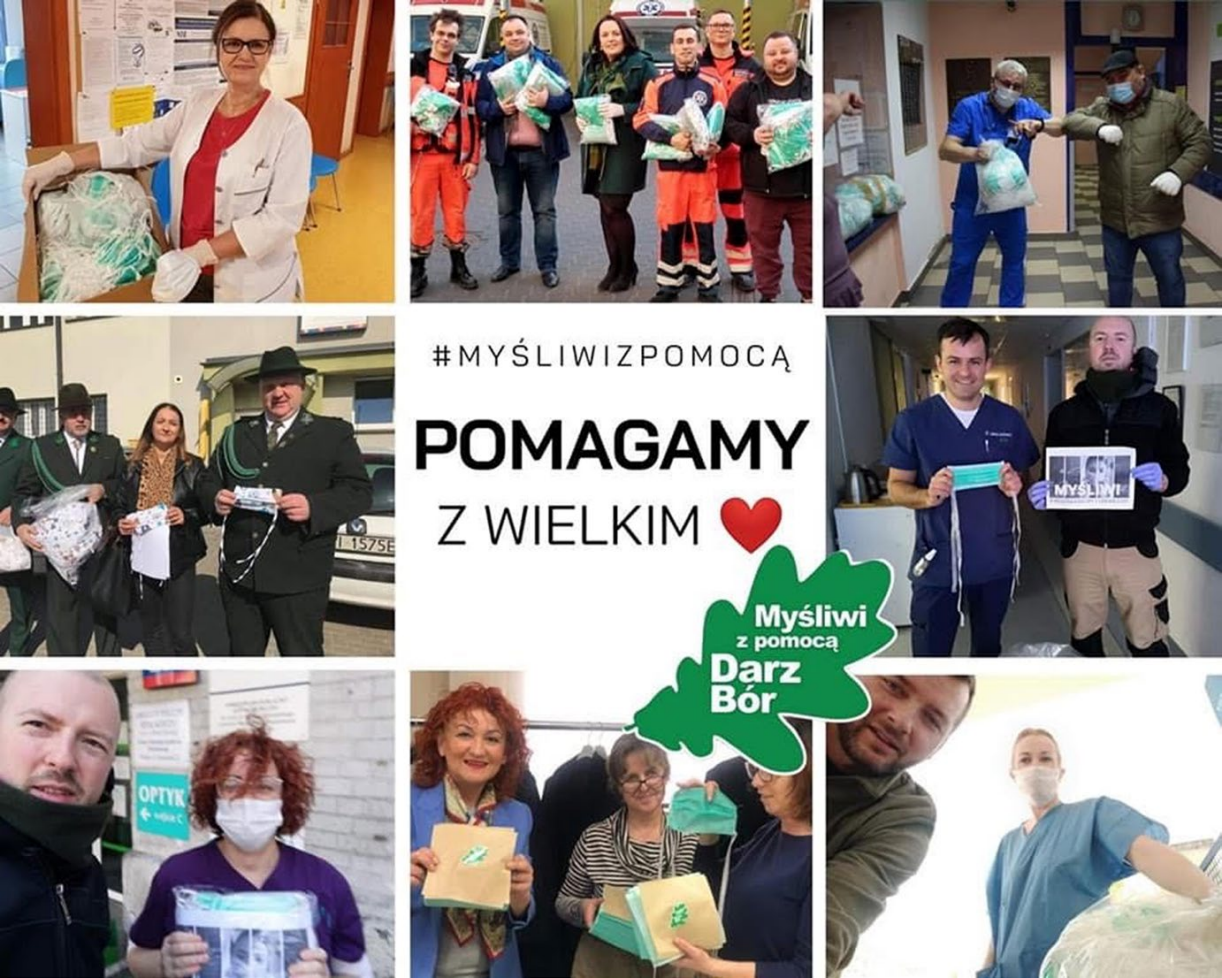
Hunters with help action

**Fig. 2:**
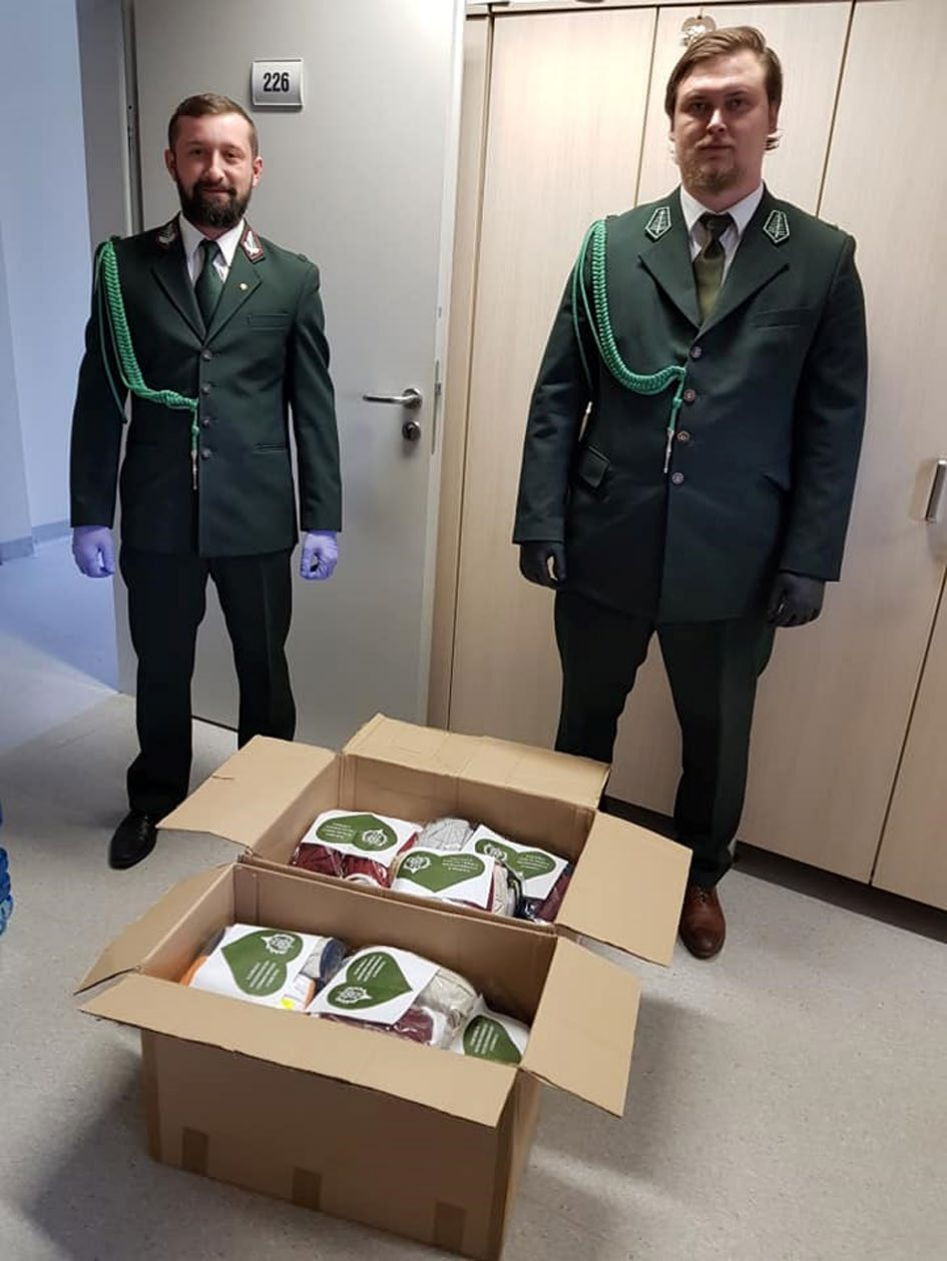
Venison from hunters to medical and paramedical staff

In Poland several charity actions were organized spontaneously by non-charity organizations. For instance, Polish hunters organized several actions. One of them was the operation of sewing masks from attested materials for hospitals. The hunters organized money collections online and bought attested fabric. They contacted tailor shops to have the masks sewn. They also delivered water and venison products to hospitals. According to the information published at the website of the Polish Hunting Association over 350,000 medical face masks, 20,000 face shields, antibacterial and antivirus liquids, disinfectants and other equipment (including protective clothing) as well as food (venison products and water) (data from 11th May 2020) (PZŁ in Szczecin) were delivered to hospitals. The total value of delivered goods amounted to PLN2 mln (EUR 500,000). Hunters-volunteers took part in the action “Hunters with help” and distributed over 180,000 masks to hospitals and social care homes throughout the country. They also bought over 2000 face shields for medics (Figs. 1, 2).

**Fig. 3:**
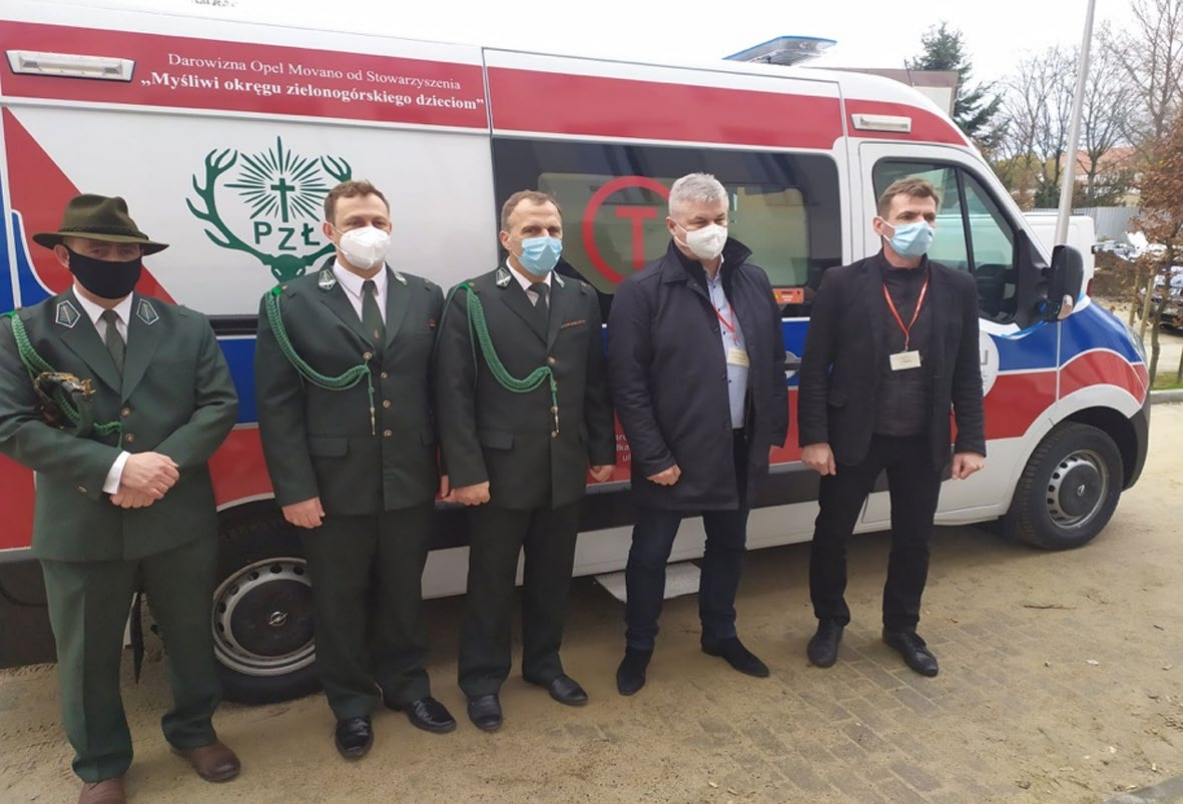
The ambulance financed by the Association of Hunters from the Region of Zielona Góra

The Association of Hunters from the Region of Zielona Góra, Poland, bought and equipped an ambulance for one of the hospitals to transport patients (Fig. 3).

**Fig. 4:**
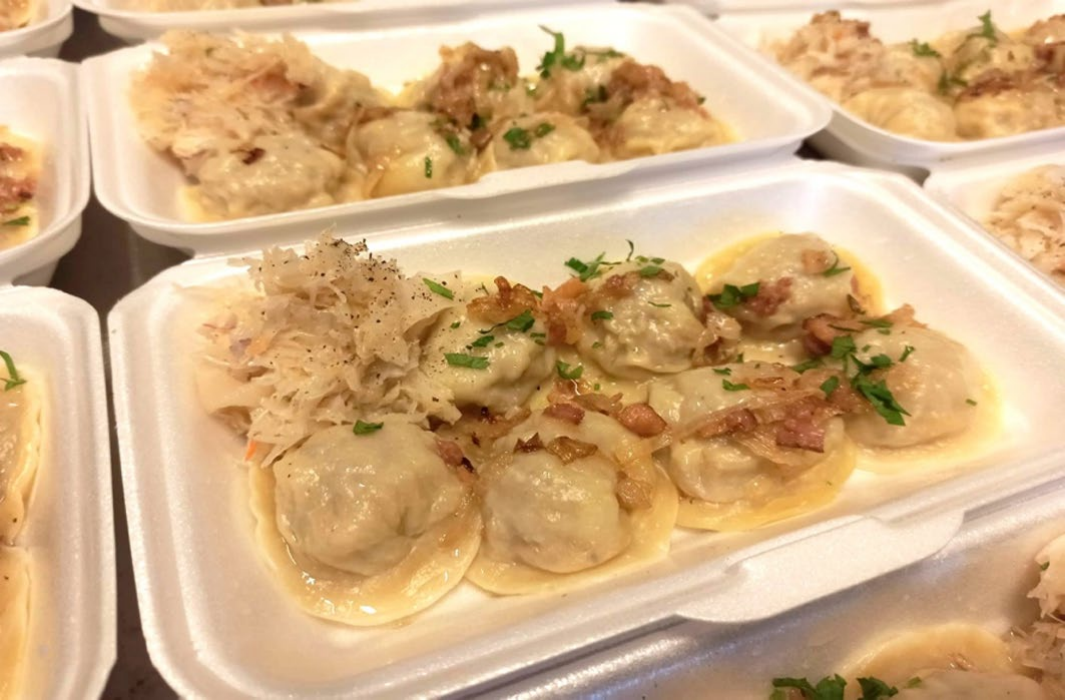
Meals prepared by the restaurant “At hunters” in Poznań and Polish Association of Hunting—Poznań District for staff of the hospital in the City of Poznań, Poland

Some restaurants also delivered meals to hospitals. The action was organized under the banner “Meals for medical staff”. The restaurant and pub owners were very heavily affected by the Covid-19 pandemic as they were not allowed to sell food and drinks on site. They were only allowed to provide food on the go. The charity action was a method of not throwing away already bought products and trying to save jobs. The second wave of restrictions introduced in the autumn of 2020 was much harder with many firms already liquidated or at the verge of insolvency. Therefore, the charity actions could not be offered at such a large scale as in the spring of 2020 (Fig. 4).

In the autumn 2020 the Polish government, however, prepared the act giving increasing remuneration to medical and paramedical professions involved in combating Covid-19 pandemic. The pays were supposed to be doubled. Though as the legislation process was hectic they made a mistake increasing pays to all medical and paramedical professions. As a result, the law was not published in the Official Journal of Laws on 28 October 2020. The legislator decided to amend the unpublished law and publish it together with the amendment. The amended law was finally enacted and published in the Official Journal of Laws on 28 November 2020 [31].^5^

In the United States, medical workers have been deemed essential workers. The distinction between essential versus non-essential came about early in the pandemic in terms of those businesses that could remain open to the public and those that had to shutter their doors [144]. Millions lost their jobs and unemployment rates skyrocketed. And, even while frontline medical workers have been applauded for their selfless, tireless work in treating patients, many nonetheless experience violence insofar as their bodies are the bodies most at risk of infection and subsequent contagion [145].

### Docile Individual Body

Partitioning individual bodies was highly essential to curb SARS-CoV-2. A moral, or even, a moralistic discourse was set up to impose restrictions on free movement to individual bodies that were said to be not useful for the countries during the pandemic. This category of people is named: docile individual bodies. The moral discourse has been internalized by individual bodies, leading to self-surveillance and self-restrictions, making them disappear from the public sphere to abide by the regulations. So, this encompassing gaze of power emanates from the television and individual surveillance practices. Docile individual bodies are disciplined ones: they self-control, self-regulate and self-restrain their habits during and after SARS-CoV-2 pandemic, and even denounce other individual bodies who are less docile in their view, with or without proof.

During the pandemic, docile individual bodies were obliged to stay at home, and were subject to significant mobility limitations to prevent the first category (i.e., “useful individual bodies”) from being further overwhelmed by the massive influx of patients. Thus, these bodies were officially locked up by regulating authorities in their homes to prevent the pandemic from increasing. For a long time, restrictive administrative authorities have limited civilian unmanned aerial vehicles (UAVs) to a few specific uses, amongst others film shooting, treatment of agricultural land, or cartographic surveys. But all changed with the outbreak of SARS-CoV-2 pandemic. Over a certain period of time, flying robots appeared in several countries, like South Korea [85], Germany [74], the UK [113], France [47], Spain [114], Italy [119]. Authorities used them to disseminate warning messages, detect suspicious movements in the streets or flush out illegal gatherings. In Poland, drones were used to transport samples for examination between a temporary hospital in Warszawa and the main Covid-19 hospital laboratory.

This time period was really so stressful for docile individual bodies that some police officers in some cities took initiatives with parodies of famous songs to relieve people from their stress and incite them to stay at home, for instance in Catalonia—Spain [49] and Brussels-Belgium [32]. Even famous singers performed re-interpretations and/or parodies of hits, explaining the lockdown [117], asking to comply with sanitary rules [79, 131, 138] and/or thanking useful bodies that sacrifice themselves for the good of others without any consideration for their own safety [87]. In Poland, artists started recording songs encouraging people to stay at home during lockdown. The songs were directed at various strata of society. Some of them had very blunt lyrics, sometimes even with vulgar or slang language, e.g.: the songs by (1) “Freeborn brothers” titled “Siedź do chuja w domu” (Sit the fuck at home) [70], (2) “Big cyc” group titled “Ja chcę leżeć” (I want to lie down) [38]. There are also songs for kids encouraging them to wash their hands properly and take care of hygiene in the coronavirus pandemic time, e.g.: Koronawirusa przegonimi (We will chase away the Coronavirus) [95]. Famous and ordinary citizens joined the rapping challenge for a Covid time song that flooded the social media such as Facebook in spring 2020. The artists from various regions also cooperated and recorded songs, for instance to thank medics and paramedics for their work [92]. The spontaneous social campaign “Siedź na dupie” [Sit on your ass] inspired artists, politicians and scientists to publish a wide variety of materials aiming at encouraging people to stay at home and keep social distance. Numerous mems were also created for those purposes.

Docile individual bodies then reinvented themselves during the lockdown, creating new models for networking with online aperitifs and meal sharing. While the human aspect was overshadowed, the openness to others was accentuated. Television was also a driving force in networking between lambda people and celebrities through games, cooking classes and gymnastics. Even docile individual bodies decided to help and used their talents to propagate messages, as in the Middles Ages streets criers used to do. These initiatives were aimed at giving news of neighbors and family to relatives who were isolated in their homes, but also allow exchanges to be set up to provide a way to enjoy this time of compulsory seclusion [62, 67]. To further avoid the psychological distress surrounding the virus, the authorities also broadcast popular, soft, cult films that were supposed to make people forget the confinement for 2 or 3 h,^6^ and they decided to reschedule violent films, hospital series later on.

**Fig. 5:**
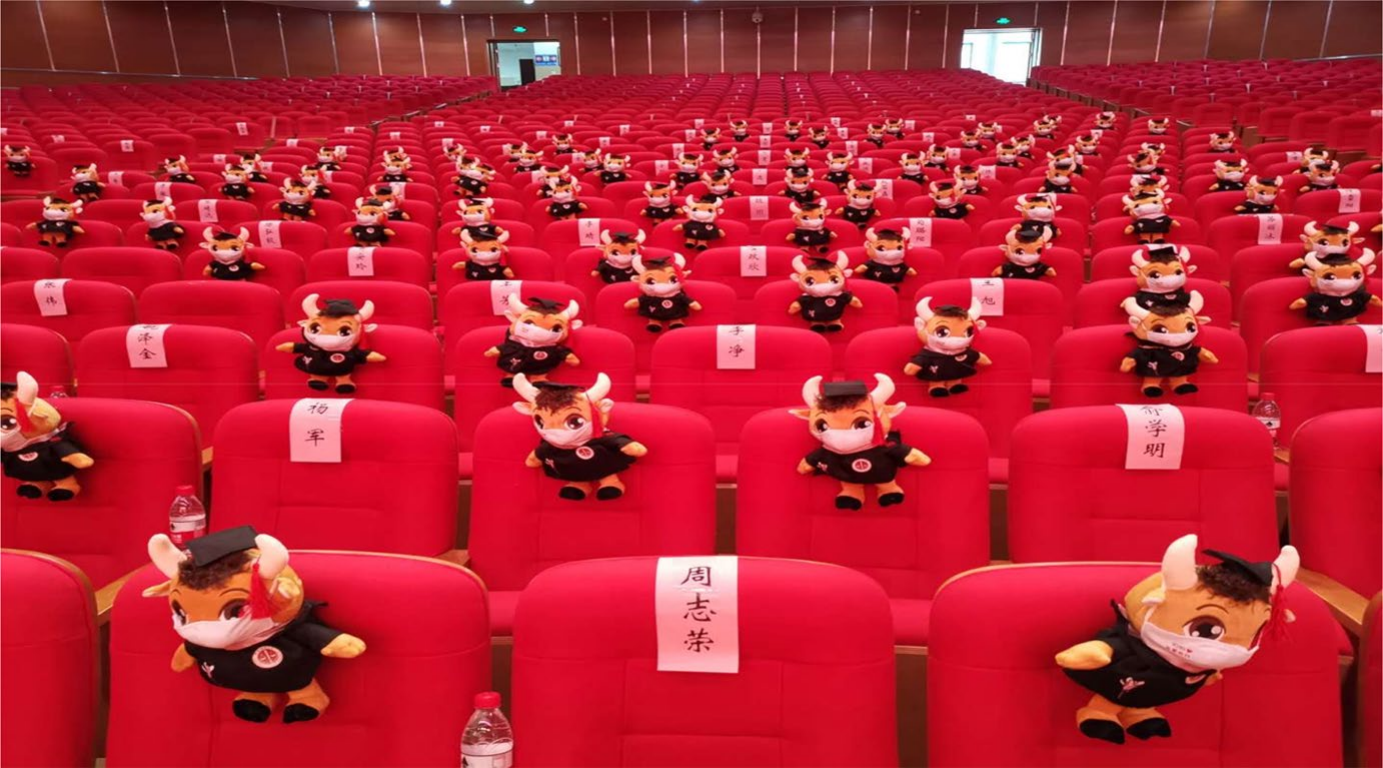
Graduation Ceremony at Zhejiang University—23 July 2020 (Respect of Social Distancing) (The official puppet is called “Tuo4huang1 Ox”, in Chinese “拓荒牛”)

**Fig. 6:**
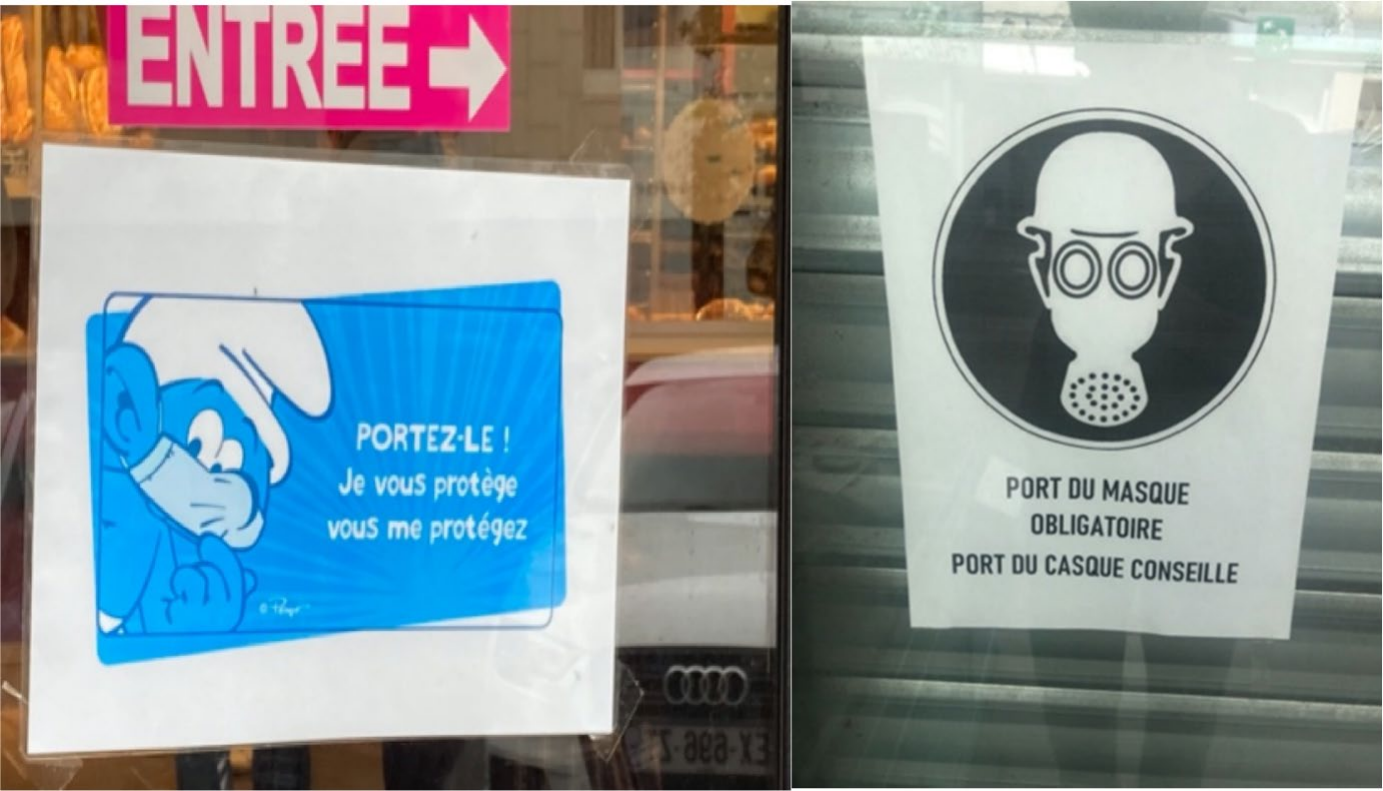
Shops in Boulogne-sur-Mer, France (respect of face covering)

**Fig. 7:**
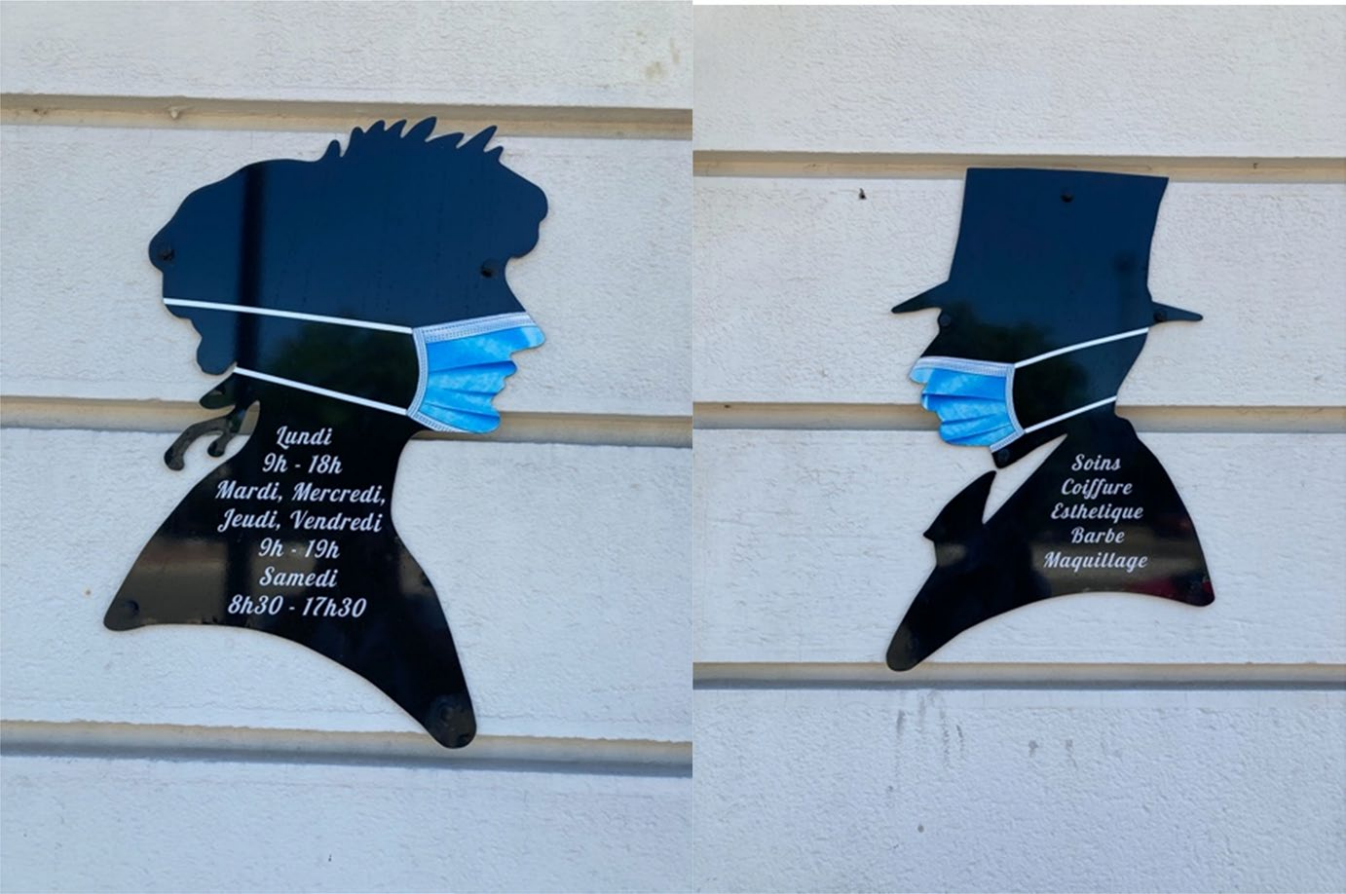
Haidresser’s front door in Boulogne-sur-Mer, France (respect of face covering)

Necessity being the law, no space escaped this systematic surveillance. When the confinement was lifted, rules were more flexible and health protocols were clarified to the population: social distancing, hand washing, and wearing of facemasks. The aim was to put the economies back in motion, to allow the population to move out with specific rules, and above all to ensure that the various social activities could resume again. To ensure the protection of all, rules were put in place so that even if mobility was once again allowed, it would remain controlled through signs on the ground, painted circles in playgrounds [133], official puppets in graduation ceremonies (see Fig. 5), displays on store windows (see Fig. 6) and at the hairdresser (see Fig. 7), among others.

Even beaches were regulated, prioritizing mobility over immobility, and when immobility was permitted, it was visible by stakes indicating where and how many people could gather there [102]. In addition, people needed to book in advance to have access to some beaches [42]. Some cities decided to limit access to beaches when the tide was high, thus ensuring that social distancing was maintained, while others were even more restrictive, closing the beaches at the end-of-day to avoid evening crowds that would result in unauthorized beach parties. This was also the case in some public gardens, particularly in New York [45].

In Poland, public parks were closed to the public in spring 2020. In the fall, the Polish government did not dare to close them for fear of social riots. However, various restrictions were introduced limiting the number of participants and audience of sports, theater, cinema and other entertainment events and concerts. The limits at various stages ranged from selling 75%, 50%, 25% of all available seats to a total ban. At the periods of full bans, for instance sports events could be organized and broadcasted without any spectators in stadiums. Major employers in regions started organizing help for their employees who for a variety of reasons could not leave homes. For instance, the Adam Mickiewicz University in Poznań (Poland) organized the action “A Visible Hand” (Widzialna Ręka) [60]. Employees from various districts of the city were asked to contact the help center. Volunteers from the same district were given a list and contact data of the needy ones. They were given the task of doing shopping for them up to PLN200 (about EUR45). The money was preferably returned via bank transfers, and the shopping left at the doormats to avoid direct contact between volunteers and the needy ones. They have also organized the collection of goods needed by one of the Poznań hospitals.

The rapidly increasing number of people diagnosed with Covid-19 and quarantined paralyzed health systems in many countries. The overcrowded hospitals turned out to be unable to treat patients due to staff shortages. In Poland, forest rangers from the forest districts of the Regional Directorate of State Forests, volunteered to help the University Clinical Hospital of the Military Medical Academy in Łódź. The foresters underwent training in operating oxygen supplying equipment and were delegated to the hospital where there was only one worker able to do the task [100]. Thus, the docile bodies of foresters transformed into useful bodies.

In the United States, bodies that travel are both conflicted between being useful and harmful. Those who travel serve as the basis for tourist-based economies, such as Hawai‘i. Tourist bodies are bodies split between the freedom of individual travel and the reality of group rejection in terms of targeted quarantine restrictions and testing mandates that attempt to prove such bodies are without pathogen. The docility of the tourist in the age of Covid-19 has transformed from one of individualized leisure [8] to one of juxtapositionality between potential viral spread and presumed community threat to economic buttress and much-needed driver of economic stimulus [146].

### Conflicting Individual Body

When dealing with power, another group of individual bodies arise, not ready to comply with any external rules imposed on them. This category is composed of conflicting individual bodies, who always deny power from the State and who only want to remain independent and free of any subjugation. This category of people is certainly the most difficult one to manage as their consideration is radically distinctive from the common interests of the country. Conflicting individual bodies always view power as manipulative, coercive, and so as a threat to their freedom of mobility and of speech.

From time immemorial, certain bodies have challenged the authorities in charge of compliance with health measures, prioritizing their own interests over the common interest. This was the case of Typhoid Mary in New York in early twentieth Century. She showed no symptoms of typhoid, but with her work as a cook for affluent families, she disseminated the disease, without knowing she was a carrier of it, infecting and sickening many people. She was identified as patient zero, but as she willfully changed her name and so refused to stop her activities, health authorities decided to isolate and lock her up for the rest of her life in a containment site on New York’s East River [10], where she ultimately died, vilified by the whole population for her irresponsibility towards others. In fact, this is the first known case of civil disobedience.

This movement of civil disobedience has been visible during and after the pandemic outbreak. Under more or less scientific pretexts, some citizens are breaking free from the chains imposed by the authorities, defying the prohibitions that have now been imposed on them. This category of conflicting bodies refuses to be confined to a given space defined by others, and seeks to emphasize its freedom of mobility against general sanitary rules. There are those who act the invisible so as not to be detected, and those who act the visible so as to be identified.

It all started when governments quickly decided to confine populations. These conflicting bodies then chose to flee the small studios or apartments they have in cities (primary residences) to take refuge in their second homes or vacation homes, therefore increasing the population in some seaside resorts without warning, and rising problems of delivery for common necessities [51, 59, 65, 120]. After the confinement was lifted on 11 May 2020 in France, other rules were imposed such as the limitation of mobility being restricted to 100 km around the main residence. These conflicting individual bodies can still now be fined 135 euros. In the event of a repeated offence within 15 days, the fine could increase to 1500 euros. After four breaches within 30 days, the offence becomes a misdemeanor punishable by a fine of 3700 euros and a prison sentence of up to 6 months. But nothing succeeds in preventing this group from acting according to its own interests. Accordingly, conflicting individual bodies further braved the bans during the lockdown, wanting at any cost to enjoy well-deserved vacations, and so they drove at night on small country roads to reach their holiday resorts (Spain, Wales, etc.). Some of them were stopped and ordered to drive back after paying a high fine, others arrived safely [41].

Eventually, civil disobedience increased even more when the governments decided, after the lockdown period, to mandate the wearing of masks for all outings, especially when the number of people did not allow social distancing to be maintained, knowing that droplets are sprayed into the air from the moment infected people talk, cough and sneeze, and so possibly leading to SARS-CoV-2 outbreak. A part of the population had a countermovement to restore its freedom of mobility, despite effective bans, generating some unavoidable conflicts with the forces of law and order. This is what happened in France, for instance, on August 2020. A banned rave party in Lozère brought together from 7000 to 10,000 conflicting individual bodies on an agricultural field in the Cévennes National Park, where they had gathered illegally. This was done in spite of the maximum gathering gauge set by the French authorities at 5000 people to limit the spread of the virus. Judicial authorities have now opened an investigation into the organization of an undeclared and illegal event in the time of Covid-19, and the equipment has been seized. But smaller conflicts with far fewer conflicting individual bodies also took place in tourist areas, namely on the beach. In Belgium, on August 9, 2020, a general fight broke out, after a group of conflicting individual bodies refused to apply sanitary regulations. The violent brawl involving several dozen people occurred when Belgian authorities asked the group to leave the beach. Individuals used beach sunshades as weapons against police officers. For this reason, verbal and physical aggression is on the rise as a direct consequence of the pandemic, with some conflicting individual bodies believing that their fundamental rights are being violated, while docile individual bodies consider that the health rules imposed by governments must be enforced. These conflicting individual bodies challenge the authorities, and so even bus drivers could be faced with more or less violent reactions from passengers reluctant to apply the sanitary rules. This is what happened on July 2020 in France, when a French bus driver asked passengers to wear their facemasks. He was beaten to death [68]. But violence is not always as intense, and can lead to verbal clashes with store security guards in charge of enforcing the sanitary rules when entering the shops (i.e. masks and sanitizing gels). Shops have to constantly solve the problem of disobedient customers who do not want to put on a mask. In Poland there were a few unpleasant incidents (offences) with customers attacking physically shop assistants who refused to serve them because of the lack of the mask on their faces. One of such customers was sentenced to two months in jail. The same customer behaved aggressively at previous visits in the shop. He offended the shop assistant and uttered threats against her [134]. Several flights had to be postponed because passengers did not want to sit in masks on board the plane [40]. Similar incidents have been reported in buses and trains [88, 107]. Usually such “rough boys” are fined. Some conflicting individual bodies, believing that they do not represent the police force, disregard their recommendations.

In the United States, there is no national mandate for mask-wearing during this pandemic. Only thirty-eight states, plus the District of Columbia and Commonwealth of Puerto Rico have state-based mandates to wear a mask [147]. Because the United States is geographically focused on the continent of North America, with the exceptions of two states (Alaska and Hawai‘i) and U.S. Territories (Puerto Rico, American Samoa, Guam, Northern Mariana Islands, and the U.S. Virgin Islands), travel among the 48 states is quite common, mixing those who wear masks with anti-maskers. Even while infection rates have skyrocketed, with the U.S. leading the world in viral spread and infection rates (as of January 2021) [55], the practice of wearing a mask is politically divided between Republican and Democrat. This violent distinction was made evident to the world during the violent siege of the U.S. Capitol by pro-Trump, white supremacist domestic terrorists as well as during the lockdown of legislators [148].

There is another category of conflicting individual bodies, who wants to be heard, detected and known. This category includes anti-maskers who believe that their fundamental rights and freedom of mobility are being challenged by the competent authorities. Hence, they organize large demonstrations to show their opposition, and their desire for independence and autonomy, with groupings far exceeding the authorized number of people allowed to gather (almost 18,000 coronavirus skeptics in Berlin [48, 69]), with non-compliance of wearing masks/face coverings and social distancing [75]. Some believe it is a coronavirus conspiracy theory, which was exaggerated and invented to exercise full control over people [61]. This theory escalated even more, esp. in the US, when President Donald Trump retweeted a fake video about an anti-malaria drug being a cure for the virus [82]. President Bolsonaro in Brazil also backed this treatment [33]. Facebook and Twitter reacted quickly and blocked videos from Brazilian President for SARS-CoV-2 misinformation [84] and Donald Trump for his election campaign [122].

The fact that theater performances and concerts were cancelled or took place with the limited number of spectators diminished significantly the earnings of artists. They became the conflicting individual bodies expecting help from the government and society. It resulted in a huge number of posts from artists criticizing the authorities and complaining that they cannot earn a living because of restrictions. Some of them decided to undertake other jobs. For instance, some Polish actresses started selling online hand-made products such as sweaters, scarfs, etc. Musicians offered online music classes. The owners of some facilities, mostly severely impacted by the lockdown (i.e., passenger transport firms, gyms, fitness clubs) decided to protest and evolved from docile to conflicting bodies.

Lastly, tracing the impact of bodies that come into contact with other bodies is the *raison d’être* for the implementation of contact tracing programs. Contact tracing frames the individual and his/her associations with other individuals in terms of a web. Through contact tracing, the movement of bodies is a source of data ripe for collection [149]. However, because the links between individual bodies are so complex and intricately fluid, contact tracing is an incomplete method for stopping the viral spread.

When partitioning individual bodies into three categories, governments have enforced a societal stratification within society, leading to imbalance of power and a systematic surveillance over individual bodies. However, the boundary between these three individual bodies is much more than fluid, since a docile individual body can transform itself into a useful individual body or a conflicting individual body. In practice, this original stratification, which appeared at the lockdown period, has imploded as the health situation, the restrictions imposed by government, and the individual's behavior evolve. Hence, the stratification of individual bodies into three distinct categories may change, based on the circumstances and temporal spaces in which the virus impacts the citizens' lives.

## Economy of Suspended Time and Rights

With SARS-CoV-2 outbreak, modern societies are living in an economy of suspended time, and suspended rights. So, it is more a notion of perception, of grasping this so-called suspended time, of realizing that in extreme conditions—different from those of a prison—the rights of some and others are put on hold for the benefit of a community, and that individual bodies must adapt to this new order, this new ‘pain’ [5] by putting in place resilience measures to limit the psychological impacts of such an arsenal, such security measures that surround bodies for their own protection.

This means that the ‘pain’ at the heart of punishment is not the actual sensation of pain, but the idea of pain, displeasure, inconvenience – the ‘pain’ of the idea of ‘pain’ [5, p. 94].

The theory of suspended economy can be experienced as oppressive, intrusive, and even dangerous for individual freedom and rights. Furthermore, having no determined temporal framework clearly increases notions of anxiety, as an incapacity to extricate oneself from an endless loop that began before and during the lockdown, and still now prolonged in time after the economic recovery with ever-changing regulations. Understandably, a prisoner is locked up for a determined time period and precise reasons, whereas in the current situation people are subject to the vagaries of time, the virus evolution that may impose rules proven to be radically distinctive, or even contrary, from one country to another.

In this flow of change that adapts to time and space, a growing sense of insecurity appears among people. With the theory based on suspended time and rights, variables or unknown data, may (dis)appear when the situation becomes even more complex. Typically, this theory is based on a variable geometry having some precise measurements, indefinite or unknown variables to allow new manipulations—i.e., regulations—to be set up in a time of restrictions, lockdowns, or even curfews around the world.

[they are] techniques for rationalizing and strictly economizing on a power that had to be used in the least costly way possible, thanks to a whole system of surveillance, hierarchies, inspections, bookkeeping, and reports – all the technology that can be described as the disciplinary technology of labor [6, p. 242].

In addition, States isolate other variables that are needed to curb SARS-CoV-2 outbreak with the help of scientists. As such, they simulate potential and conceivable scenarios with the help of High Performance Computing (Epidemap [66] in France, Covid-19 Dashboard by the Center for Systems Sciences and Engineering at Johns Hopkins University—USA [55]). The Covid-19 High Performance Computing Consortium [130], launched on 22 March 2020, is a cross-disciplinary initiative involving governments, industry and research institutions to bring the most advanced computing resources to accelerate the discovery of potential treatments and vaccines against SARS-CoV-2 [121].

As a result, within this economy where rights and time are suspended, the States have put in place a variety of modalities, alternating between freedom and restriction, so allowing the implementation of extraordinary measures during and after the lockdown, but also leading to some disruptions.

### An Economy of Extraordinary Measures

In view of the endless and uncontrollable virus increase, countries have put themselves in an orderly battle against this scourge. However, this scale of battle is very patchy from one country to another, posing operational problems when borders are being crossed by people from different countries, and implying a rather complex implementation of an arsenal of regulatory measures and technological devices.

The new technology that is being established is addressed to a multiplicity of men, not to the extent that they are nothing more than their individual bodies, but to the extent that they form, on the contrary, a global mass [6, pp. 242–243].

In the current state, detections and analyses of clusters in countries have led to the emergence of evolving limitations, similar to data mining in IT science, focusing on supervised global mass. These evolutionary variables lead to cluster analyses, the distance and (dis)similarities between them. Given a set of observations, regulating authorities aims at partitioning, limiting and restraining people’s mobility.

Some countries, while remaining vigilant and circumspect, have implemented restrictive measures, of varying lengths, to limit the number of potentially infected persons entering their territories. Countries around the world first closed their borders, regulating entries by banning specific countries deemed to be at risk, or by allowing their arrival on condition that they quarantine themselves. These quarantine measures were manifold, and depended on which of these countries might consider others to be on the red list, while others considered them safe. In Europe, during the coronavirus pandemic, travel restrictions were implemented to slow down SARS-CoV-2 spread. As from 15 June 2020, the European Commission launched “Re-Open EU” [118], an online platform with all the necessary information on free movement and tourism across Europe. EU member States have now adopted a recommendation with a classification of regions (green, orange, red and grey) based on the local epidemiological situation to provide timely information to European passengers [103]. Other countries worldwide decided either to suspend their international flights to Europe, to forbid or to limit the arrivals of certain passengers on their territories. Australia set up from the very beginning biosecurity measures and travel restrictions, stating that the only people who could travel to Australia were Australian citizens, residents, immediate family members, permanent residents, New Zealanders [53]. These travel restrictions are still valid on October 2020. In June 2020, the UK implemented a mandatory 14-day quarantine for all arrivals from other countries.

The European Council also has launched a website “Timeline—Council actions on COVID-19” [132], providing updated information of its actions and recommendations for Member States in response to SARS-CoV-2 pandemic. In Poland, various restrictions were in force at various stages of the pandemic including the list of countries from and to which flights were suspended, the quarantine periods or self-isolation periods for travelers, the obligations to have a Covid test done just prior to entering into Poland, Switzerland, etc. For instance, flights from Poland to China were suspended by the Civic Aviation Administration of Chinese from 2 until 30 November 2020 after finding out that 10 passengers infected by Covid-19 had been brought to China by the Polish Airlines LOT in October [116]. In spring 2020, the Polish Airlines LOT also organized rescue flight to collect citizens who got trapped in the territories of other countries (e.g. Great Britain, Malta, Cyprus, Ireland, the USA, Portugal and Canada) and could not go back to their homeland [39, 78]. The Polish government offered help to the American Embassy by taking American citizens to their homeland when flying to that country for Poles [135].

Because the virus is so rampant in the United States with increasing rates of infection and soaring death rates, many countries around the world have closed their borders to admitting American citizens. As of January 2021, Americans are restricted from Canada, countries in the European Union, and many countries in Asia [150]. Although there is no national mask-wearing mandate in the U.S., the American Embassy recognizes that the mask is worn globally and that U.S. Citizens should do the same:

U.S. citizens should be aware that failure to adhere to mask-wearing norms reflects poorly on foreign residents and may result in a fine [151].

Right now, with the second SARS-CoV-2 wave, countries set up other limitation regulations from quarantine, short-term lockdowns, partial lockdown, full lockdowns to evening curfews. It all depends on the country’s health situation, the R number [30], and how the governments want to restrict mobility and freedom on people. These decisions are now easy to implement as the first consequence will be a direct impact on the economic recovery of companies, restaurants, cafés, cinemas, etc. Contrary to the first lockdown that was mainly imposed all over the world at different periods of time, these new restrictions intervene on a wide period of time and try to restrain the impact over the economy, though it is still difficult to know in the long-term what will be the exact consequences over the economy. Furthermore, governments decided to set up recovery plans, like the Marshall plan after WWII, in order to safeguard as much as they can their economies, their Gross National Product with more or less important recession, leading to substantial unemployment rate and bankruptcies.

Israel decided on a second lockdown of 4 months starting on 18 September 2020, after the infection and mortality rates skyrocketed. Ireland is the first EU countries to lockdown again for two weeks as from 20 October 2020 due to a surge in SARS-CoV-2 cases, in the hope to save the Christmas season. Wales did similarly by imposing a national circuit-breaker lockdown from 6 pm on 22 October 2020, which will last until 9 November 2020. France set up an evening curfew, from 9 pm to 6 am, as from 25 October 2020 for a period of 6 weeks before deciding how to proceed further in some specific places where the virus is increasing too fast to avoid too much tension in hospitals. As from 30 October 2020, France is confined again, but this second national lockdown is less restrictive than the first one, since schools and firms remain open. Social gathering is banned. Only universities and non-essential shops, restaurants and bars are forced to stop their activities at least until 1 December 2020. Germany imposes a partial lockdown as from 2 November 2020 till 30 November [50] with closures of cinemas, theatres, gyms, pools, restaurants and bars. Social gathering should not exceed 10 people maximum, but similar to France schools remain open as well as visits to nursing homes. Belgium is also locked down as from 1 November until 13 December 2020, with the closure of all non-essential stores. Social gathering should not exceed more than four people [123].

In Poland, on October 2020, a curfew was introduced for children and teenagers to keep them home during distance teaching hours (from 8.00 a.m. till 4 p.m.). The first group of enterprises that were locked down were gyms, fitness clubs, swimming pools and aquaparks. Restaurants and bars were forbidden to serve food and drinks inside and only allowed to sell food on the go from 24 October 2020 [108]. During the first wave of Covid-19 pandemic events, such as wedding receptions, were forbidden. Then, the ban was temporarily lifted with limits established for various zones. Initially 150, 100, 50 persons were allowed to participated in weddings in green, yellow and red zones respectively. Later on, the limits were reduced to 100 in green zones and 75 in yellow ones. From 10 October 2020 whole Poland was either in yellow or green zones. From 17 October the limits of 75 persons participating in weddings and wedding receptions as well as funerals in yellow zones and 50 persons in red zones was introduced [96]. Social gatherings were limited to the maximum amount of 5 person from 24 October 2020 [115, 140]. The ban on weddings, funeral banquets and other special events was in force from 19 October 2020 in the regions located in the red zone. From 24 October 2020, all of Poland became a red zone with the ban extended automatically to the whole territory of the Republic of Poland [43]. The audiences of cinemas, galleries, theaters and concert venues could be filled only in 25 percent from 10 October 2020. From 7 till 29 November 2020, such institutions as well as libraries were closed completely to limit the mobility of citizens as much as possible [141]. School children gradually were sent home for distance classes starting with the secondary schools from 18 October 2020 in red zones, [141] and finally with primary school students from 4th grade up were sent home on 26 October 2020, and primary school students from 1st till 3^+^ grade on 9 November 2020 [104]. The distance learning was said to last at least until 29 November 2020 but it was prolonged with the decision that only primary school kids from grades 1 to 3 allowed to come back to school starting 18 January 2021 [142].

Throughout the United States, public schools have converted to online teaching and learning. In contrast, many privately-funded schools remain with in-person learning. The consequence of an absent national policy in terms of primary and secondary education is a growing divide between the wealthy and privileged and the poor and working class. Attending in-person school for those parents who can pay for the opportunity is a statement on where the priorities are for children’s intellectual development throughout the United States. Once a strategy for equalizing education access and opportunities between social classes, public education has succumbed to the deleterious effects of Covid-19 [152]. In addition to the data collection from contact tracing methods, the intellectual growth of school-age children has become a commodity through technology companies set up to simulate, if not replace, the human teacher and classroom of peers.

Countries around the world also concentrate on mass-surveillance technologies to monitor SARS-CoV-2 [1]. They created apps to download on citizens’ smart phones in order to track, detect and isolate people positive for Covid-19. France created the app “StopCovid”, but it was not downloaded quite enough to help detect contaminated people, so the government for the second lockdown updated this app and called it “Tousanticovid”, in the hope that many French citizens will use it to trace, detect and isolate to curb the contamination chain [99]. However contrary to most European apps, the French application cannot be used outside French territory. The French government raised privacy and data protection issues, being incompatible “with regard to the nature of the data and the risks of the processing, to preserve the security of the data…" (article 34 Act of 6th January 1978, known as the act on "Information technology, Data files and Civil Liberties"—hereafter referred to as the French Data Protection and Freedoms Act or FDPFA). So far, 17 European Member States and the European Commission worked on a eHealth Network guidelines on June 2020 to set up “an interoperability specifications for cross-border transmission chains between approved apps” [63], to quote a few: Corono-Warn App (Germany), Immuni (Italy), Apturi Covid (Latvia), and SwissCovid (Switzerland) [72]. In Poland, the application STOP COVID ProteGO Safe was launched. The application uses Bluetooth to locate phones of quarantined and infected persons. It posts alerts allowing to distance oneself from such persons. It also enables to assess the risk of COVID infection of the application users. The application also informs about the up-to-date restrictions in force in the territory of the Republic of Poland [125].

Across the world, COVID restrictions turned out to be destructive for local economies with numerous businesses affected variedly [81] but some of them deadly. The gyms, fitness clubs, pubs, restaurants on numerous occasions were not able to survive the lockdown leaving many unemployed. The subsidies offered by states turned out to be insufficient. Freelancers such as translators, artists, cleaners were also affected badly frequently having no financial resources to support themselves and their families. The restaurants and event halls turned out to be at the verge of insolvency. Some financial issues turned out to be very controversial in the course of pandemic.

For instance, in Poland on August 2020 society protested heavily and ostracized in media members of Parliament [46, 94, 126] who wanted to pass a bill increasing their compensation. Finally, the bill was not passed. The next heavily lambasted bill was the one with subsidies to artists based on the lost earnings from previous years. The bill provided for heavy subsidies to well known, affluent artists and no help or almost none to those earning the least and in fact suffering heavily as a result of cancelled performances [91, 97, 105]. From 1 November 2020 medical and paramedical personnel fighting with Covid-19 pandemic was given extra temporary increase of their remuneration [101]. The Covid pandemic actually required the governments react quickly to the dynamic epidemiological situation. In Poland that led to a huge increase in Parliamentary acts and ministerial regulations that had to be enacted. At the same time an erroneous bill was passed by the Polish Parliament intending to double the remuneration of such medics but the wording of the act in fact increased the remuneration to all group also not involved with the Covid-19 pandemic. As a result, the publication of the act was postponed [98] to give the Parliament time to enact the amendments before the act should actually enter into force [35, 73]. The amendment was finally passed and published as mentioned above on 28 November 2020.

The budget deficits, unemployment rates, homelessness rates, number of bankruptcies and insolvencies for 2020 and 2021 all over the world will show the real scale of the effect of the Covid-19 on economies. One cannot forget about the inflation and increase in costs of goods and services. Some countries consider increasing taxes to rescue state budgets.

### Disruptions and Non-respect of Human Rights

All the bodies forming societies in the global village of modern world were affected in some way. The lockdowns and suspension of flight on some routes with ban on entry of people flying from selected destinations, especially severely affected by Covid-19 virus separated families, couples and co-workers. Bi-national couples, who got stuck in two countries at the time of most severe lockdowns, were unable to be together. Students on foreign exchange programs found themselves imprisoned in a foreign country without funds to survive after the end of the subsidized programs they had enrolled to. The lockdown of students’ dormitories left them without a roof over their heads. The canteens stopped serving meals for students as well even though the studying individual bodies may have already paid a monthly fee for them. Foreigners got stuck abroad and found themselves unable to come back home for a wide variety of reasons including emergencies such as funerals or sudden illnesses among family members. [54, 71]

Due to the mobility restrictions and restrictions concerning the number of participants allowed to take part in events many people had to postpone or even cancel weddings [57]. The fact that guests could not be invited and the family reunions could not be organized affected many individual and corporate bodies. On the one hand, individual bodies had to reorganize their lives, learn how to deal with the sudden changes of plans, stress and change of lifestyles. On the other hand, the corporate bodies had to face sudden outflow of earnings and risk of insolvencies. As a result, individual bodies of employees had to deal with the threat of unemployment and loss of earnings too.

The panopticon metaphor is especially applicable when analyzing the situation of people imprisoned in a wide variety of ways, that is to say:

(i) Individual bodies imprisoned abroad and unable to return home [106],
(ii) Passengers of cruise liners where Covid-19 was detected imprisoned on board the ships and not allowed to leave despite the consequences of being easily infected as a result of being stuck in a confined space with people already suffering from the disease [37],
(iii) Inhabitants of blocks of flats without private gardens imprisoned in their apartments without access to nature, sports facilities and other forms of recreation [139],
(iv) Quarantined and isolated people imprisoned in isolation wards of hospital and their places of residence [64, 76, 124]
(v) Homeless people in most countries imprisoned in social and political paralysis [77],
(vi) Minor bodies of children who were sent home for distance learning classes imprisoned at homes without possibility to interact face to face with their schoolmates and friends. [44, 93, 111]

Individual bodies benefiting on their daily basis from a wide variety of facilities such as libraries, cinemas, theaters, concert halls, gyms, swimming pools, ice-rinks found themselves cut out of the entertainment and recreation that constituted an important part of their lives. Patients also found themselves in a precarious or even dangerous situation. Medical issue disruptions included postponed or cancelled therapies, rehabs, surgeries. The access to medical services has been limited significantly with doctors not being available to patients. The system of phone and Internet consultations started evolving. On the one hand, some people, living far away from health facilities, benefited as e-prescriptions and e-consultations were offered on a large scale. On the other hand, in the event of people suffering from illnesses hard to diagnose without examining the patient on site, the time needed to get professional help prolonged significantly and sometimes no satisfactory assistance had been offered. Individual bodies had to deal with the paralysis of the medical systems and systems of care for the elderly.

The closed borders and required medial checks that entitled people to enter a given country limited commuting to work abroad among people living in border territories. For instance, Germans complained that Polish doctors and nurses who were living on a daily basis in Poland but commuted to work in Germany could not continue working. Polish nurses and day care guardians who could not commute to Germany or decided not to commute, had to abandon their proteges overnight.

Individual bodies of teachers were expected to learn new skills to carry out distant learning courses. The first outbreak of the pandemic showed which schools were able to acquire new software, offer training and discipline their teachers to learn new skills. Individual bodies of teachers frequently opposed and rebelled against learning how to use software to communicate with schoolkids from the distance. Some schools have cancelled teaching or simply started sending class assignments via emails. As a result, individual minor bodies of schoolkids had been left alone [110]. They were expected to teach themselves instead of being taught. School teaching disruptions resulted in increased rates of depressions, domestic violence, suicides and stratification of course participants in terms of progress made [111]. The schoolkids who were more gifted or who received help from their parents would learn, whereas the less gifted with parents not able or not willing to help them had to face the deterioration of their level of knowledge instead of progress. That actually resulted in frustrations, lower marks and other long-term consequences. There were even minor bodies of schoolkids who did nothing for a few months, as parents would complete homework assignments for them. Individual bodies deprived of work and means of supporting themselves and their families were affected too. For instance, the suicide rate of women increased rapidly due to Covid pandemic in some countries (like in Japan [137]).

Many individual bodies had to face numerous health problems connected with mobility limits. General health deterioration was reported by the health community. People deprived of the possibility to exercise on a regular basis due to closed gyms, fitness clubs, swimming pools, started suffering from obesity and overweight problems. That in fact resulted in blood-pressure problems as well in some cases. The stress levels increased consequently as the medical findings clearly suggested that co-existent illnesses that result in a heavy death toll of SARS-CoV-2 include not only respiratory system problems but also obesity, overweight and high blood pressure [136].

Various curfews and penalties for breaching them immobilized bodies on an unprecedented scale. Some countries, like France, Poland, introduced shopping hours reserved solely for seniors. It meant that from 10.00 a.m. till 12.00 only people over 65 years old were allowed to enter shops. Others were not allowed to be served even if the shops were empty. Shops serving younger persons were risking paying high financial penalties. At the same time for some shops the curfew turned out to be devastating due to the loss of some customers. Individual bodies of shop assistants were imprisoned in empty shops behind glass or plex screens, wearing masks and waiting for customers.

Individual bodies having no access to modern communication means such as computers, smartphones, tablets, etc., not well versed in or absolutely ignorant about electronic communication were imprisoned in their non-digital worlds and deprived of daily routines, access to goods and services and contact with the outside world. In some countries, curfews forced people to change their daily routines. Dog owners had to adjust walks with their pupils to curfews. Therefore, many individual and corporate bodies found their constitutional liberties infringed in the state of pandemic.

## Conclusion

The modern panopticon has turned out to be the projection of future presented in science fiction movies of 70’s, 80’s and 90’s of the twentieth century. The states found out that citizens are equipped with smartphones that enable to trace and surveille them on a daily basis. Satellites and street cameras allow to identify passersby on the streets. The only things the democratic states lack is the legislation that would allow the authorities to take advantage of the modern state of the art. Therefore, the governments had to resort to persuasion and threats to force bodies to behave in prescribed and recommended manner. It turned out that bodies in some countries were more willing to cooperate with their governments but others were less willing or even rebelled against limitations.

When persuasion becomes imposition, when civil liberties and freedom are forgotten and an imposition becomes a norm to save us, some adapt whereas others fight for the lost privileges. The democratic state transforms into a police state trying to save citizens from themselves. At the same time citizens aware of the past actions of that sort, want to prevent the history from repeating itself.

The conflicting individual bodies claim all the time that the government is incompetent and violates the laws and civic freedoms. All bodies have discovered that:

*“Life was so beautiful*

*Then we all got locked down*

*Feel like a ghost*

*Living in a ghost town”* (Rolling Stones).

The ghost town metaphor very accurately depicts (1) the haunting distance people were forced to keep, (2) the haunting empty seats at the tables during important family events such as birthdays, holidays, funerals, weddings, (3) the haunting emptiness of shops and other facilities rendering services to common people, (4) the haunting empty and dark buildings of gyms, swimming-pools, concert halls, theaters and cinemas.

The generation gap between self-isolating computer fans and other members of society increased as well. Some turned out to enjoy the immobility and imprisonment at their homes. The fact that they could start working distant from their homes, turned out to be a blessing allowing them to save time and energy spent on commuting to work. For others, it turned out to be a disaster or a highly frustrating experience. The latter had to face being made redundant or copying with working with family members at home. They were deprived of their sterile work environment and had to face the challenge of teaching family members stuck together at home to respect their working-time and limits.

Some professions turned out to be especially vulnerable. The Maslov’s pyramid of needs again turned out to be the law of social co-existence. In times of disasters and pandemic individual bodies focus on their basis needs resigning for a wide variety of reasons from luxuries and privileges. The Consumer Spending Indices show the drop in spending due to Covid-19 pandemic. The reasons may be financial, health-related, enforced by law, incited by fear for endangering ones, well-being in a broad sense or threat of penalties. Eerily forecasting of today’s events, the Rolling Stones sing about the death of show business related spots:

*“Once this place was hummin'*

*And the air was full of drummin'*

*The sound of cymbals crashin'*

*Glasses were all smashin'*

*Trumpets were all screamin'*

*Saxophones were blarin'*

*Nobody was carin'*

*If it's day or not”*

That daunting dystopian reality created by the pandemic of coronavirus SARS-CoV-2 has changed the world on an unprecedented scale, forcing individual and collective bodies to adapt and fight for survival in a local prison of challenges, with limited opportunities and numerous threats. The gap between useful, docile and conflicting bodies deepens. The useful bodies risk their lives, face ostracism as they may be the virus carriers, are blessed and cursed at the same time. They are now criticized for doing too little, for being insensitive, cold and withdrawn. They are thanked for their cool heads, efforts and extra hours spend on duty. The docile bodies subordinate themselves to recommendations and orders, adapt to the life in a modern panopticon and accept the immobility. They do it, because they can. The conflicting bodies relegated to the sidelines of economic, social and family lives either rebel against inequality or give up and try to persevere. Those whose survival is threatened must oppose, fight and make their struggle visible. Otherwise, they will be buried alive under the debris of former lives destroyed by the coronavirus SARS-CoV-2.

## Footnotes

1 In French: « Une attestation de déplacement dérogatoire».

2 A lump-sum fine of 135 euros for the first offence (up to 375 euros for non-payment within 45 days), a fine of 1500 euros for a subsequent offence within 15 days and up to a fine of 3750 euros and six months' imprisonment in the event of multiple offences within a period of 30 days. The suspension of the driving licence may also be applied.

3 *Definition of a support bubble:* A support bubble is a close support network between a household with only one adult in the home (known as a single-adult household) and one other household of any size. This is called making a ‘support bubble’. Once you’re in a support bubble, you can think of yourself as being in a single household with people from the other household. It means you can have close contact with that household as if they were members of your own household. Once you make a support bubble, you should not change who is in your bubble.

4 Morphée, the reanimation module, allows to transport in a military aircraft and under appropriate medical conditions of care between six to twelve patients in a serious state.

5 Act of October 28, 2020 amending the act amending certain acts in connection with counteracting crisis situations related to the outbreak of COVID-19.

6 In France, they broadcast some of the most notorious films of Louis de Funès: Les Aventures de Rabbi Jacob, l’Aile ou la Cuisse, l’Avare, Hibernatus, amongst others.
